# From Ridge 2 Reef: An interdisciplinary model for training the next generation of environmental problem solvers

**DOI:** 10.1371/journal.pone.0314755

**Published:** 2024-12-19

**Authors:** Raechel J. Hill, Matea A. Djokic, Andrea Anderson, Kristin Barbour, Amanda M. Coleman, Alexis D. Guerra, Courtney Hunt, Amber Jolly, Jennifer J. Long, Kyle T. Manley, Jonathan L. Montoya, Carl A. Norlen, Andie Nugent-Suratt, Kameko Washburn, Samuel Weber, Allison Welch, Cynthia Wong, Steven D. Allison

**Affiliations:** 1 Department of Ecology & Evolutionary Biology, University of California, Irvine, California, United States of America; 2 Sonoma Technology, Inc., Petaluma, California, United States of America; 3 School of Biological Sciences, University of California, Irvine, California, United States of America; 4 Environmental Health Sciences, University of California, Irvine, California, United States of America; 5 Center for Environmental Biology, University of California, Irvine, California, United States of America; 6 Department of Earth System Science, University of California, Irvine, California, United States of America; 7 School of Education, University of California, Irvine, California, United States of America; 8 Department of Population Health and Disease Prevention, University of California, Irvine, California, United States of America; 9 Department of Chemistry, University of California, Irvine, California, United States of America; Johnson & Johnson MedTech, UNITED STATES OF AMERICA

## Abstract

Regional and global environmental challenges have become increasingly complex and require broader solutions than a single discipline can provide. Although there is a growing need for interdisciplinary research, many graduate education programs still train students within the confines of a particular discipline or specialty. The Ridge 2 Reef research traineeship program at the University of California, Irvine, aimed to provide transferable and interdisciplinary skill training to prepare graduate students from different disciplines to address current and future environmental challenges. The program achieved its goals through a ‘culture of improvement’ that ensured trainee needs shaped program management and curriculum. Due to trainee feedback and leaders dedicated to program improvement, there was a complete course overhaul during the first two years of the program, resulting in a final curriculum structure that was more effective and aligned with revised program goals. Program evaluations suggest that the flexibility of the program, diversity of training, overhauled courses offered, and partnership-focused opportunities contributed to more confident graduate students who were more broadly trained and better prepared in their chosen environmental career paths. Based on evaluation surveys, graduate students reported significant gains in scientific, technical, and career knowledge as well as transferrable skills in communication, data analysis, leadership, mentoring, and interdisciplinary collaboration. The structure and evolution of the Ridge 2 Reef traineeship can provide a framework for other graduate education programs to better incorporate interdisciplinary training and student feedback, ultimately improving programs and preparing scientists for the 21^st^ century workforce.

## Introduction

Science-based solutions to environmental and other societal problems have historically been limited by disciplinary boundaries that inhibit systems-level analysis [1–3]. Because they offer educational programs in a diversity of fields, universities are uniquely positioned to span these boundaries and train an interdisciplinary workforce [4, 5]. However, many educational programs at the graduate level still focus on in-depth training in a single discipline. By neglecting interdisciplinary training in curricula and degree requirements, traditional masters and doctoral programs make it tedious for students to seek collaborations or skill development outside their explicit field of study [6].

Whereas many job sectors are needed to solve environmental problems, graduate programs have typically focused on preparing students for careers in academia. This narrow focus does not reflect the post-graduate reality, in which 67% of Ph.D. recipients pursue careers outside of academia, according to the 2022 Survey of Earned Doctorates by the National Center for Science and Engineering Statistics [7]. Without sufficient training in communication, project and team management, budgeting, leadership, stakeholder engagement, and other transferrable skills, graduate students may be ill-equipped to address and resolve real-world problems that span multiple disciplines and require solution-based research [8]. Despite these urgent needs, there is little evidence-based research on how to design graduate programs that prepare students to enter a diverse 21^st^ century workforce [9, 10].

To address this knowledge gap, the Ridge 2 Reef (R2R) training program was established at the University of California, Irvine (UCI), in 2017 with funding from the US National Science Foundation. The success of the program was assessed through formative and summative evaluation over a 5-year period. The R2R program was specifically designed to train a new generation of graduate students with the skills to tackle environmental challenges, especially in regions heavily impacted by human activities. For example, the wildland-urban interface in Southern California faces threats from wildfire, invasive plants and animals, drought, extreme heat, and pollution [11]. Mitigating these threats requires collaboration across disciplines such as engineering, ecology, and law, as well as engagement with stakeholders including government agencies, private entities, universities, and the public [12].

**Fig 1:**
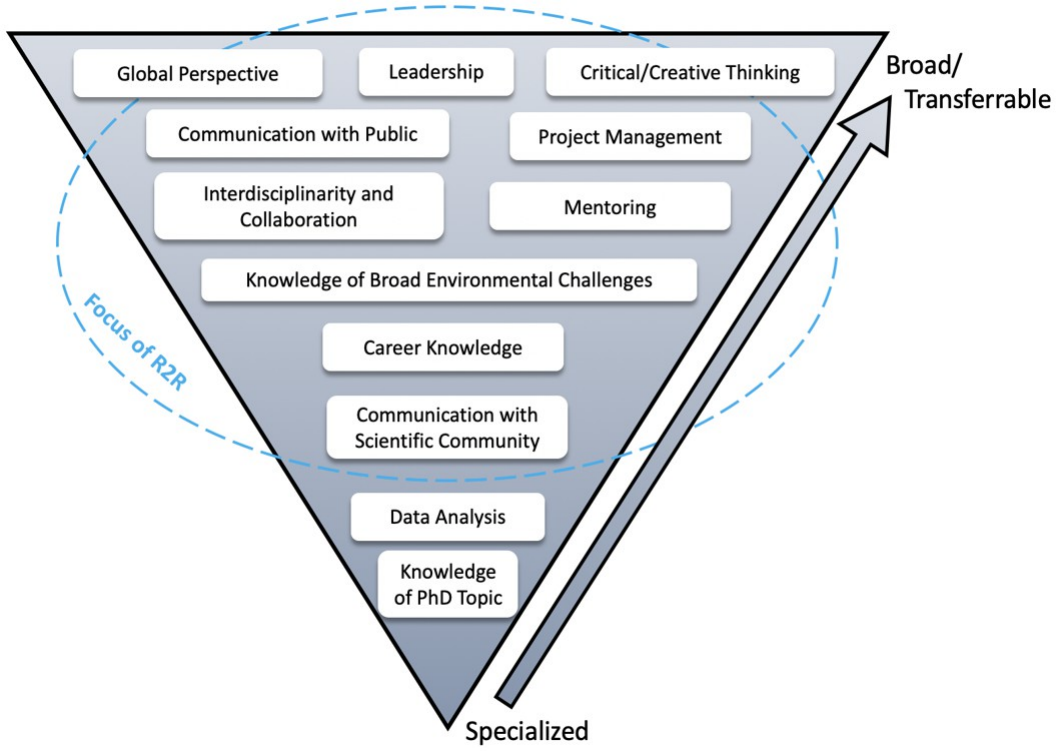
Conceptual framework for Ridge 2 Reef goals, adapted from the T-framework [15, 16].

Compared to traditional graduate programs that focus on disciplinary training and academic career paths, R2R was designed to equip students with the transferrable skills needed to pursue careers in nonprofit, government, industry, and academic sectors (Fig 1). The program aimed to develop these skills while offering opportunities to pursue projects that traditional graduate programs do not have the resources to support. With this design, R2R sought to advance students along the continuum from disciplinary to interdisciplinary and transdisciplinary thinking [3, 13]. By facilitating co-creation of knowledge across sectors, transdisciplinary approaches enable better solutions to complex problems such as climate change [2, 14]. The R2R program offered curriculum and internships to train students in solving problems across disciplinary boundaries and apply their knowledge in real-world settings. Specifically, the R2R training program sought to accomplish five goals:

- Goal 1 –*Develop scientific and technical knowledge* to facilitate management of terrestrial and aquatic ecosystems experiencing environmental change.
- Goal 2—*Promote transferable*, *career-relevant skills* through curriculum that emphasizes quantitation, communication, and professional development.
- Goal 3—*Build partnerships* in and out of academia to enhance trainee career placement and effective knowledge transfer.
- Goal 4—*Broaden participation* in the pipeline of graduates pursuing environmental careers.
- Goal 5—*Institutionalize success* by incorporating effective elements of the R2R program including courses, partnerships, collaborations, and professional development activities into other graduate programs while disseminating the training model to other institutions.

The aim of this paper is to describe the R2R program design and report on its effectiveness in achieving Goals 1 through 5 based on quantitative and qualitative evaluation methods. By reporting on the program’s design and outcomes, our paper builds on the evidence base needed for academic program leaders to develop interdisciplinary and transdisciplinary graduate training opportunities. Although R2R focused on environmental issues, the training model we developed could be applied to other fields in which graduate students must be prepared to solve complex problems, such as medicine, engineering, and social sciences. After briefly introducing the program structure, we discuss the program evaluation approach and outcomes. Finally, we discuss lessons learned that may aid other institutions seeking to build programs in transdisciplinary graduate education.

### Program description

The R2R program was funded by a $3-million grant to UCI from the National Science Foundation (NSF) Research Traineeship (NRT) program. The program began in 2017 and ended in 2023, ultimately enrolling a total of 55 graduate students (“trainees”), including 3 masters and 52 PhD students. These trainees were enrolled in five cohorts, with Cohort 1 beginning in 2017, Cohort 2 in 2018, Cohort 3 in 2019, Cohort 4 in 2020, and the final cohort, Cohort 5, beginning in 2021. Cohorts were recruited through a competitive admissions process open to graduate students working on environmental research in any discipline at UCI.

#### Governance

The governance structure of R2R included a faculty director, an academic coordinator, and an executive committee with oversight from an external advisory board. The director was the lead principal investigator on the NRT grant (Dr. Steven Allison) and chaired the executive committee. The academic coordinator built partnerships with external entities and managed daily program operations including scheduling, communications, finances, logistics, staffing, and assisting students. The executive committee advised the director and included nine UCI faculty members who were in ecology and evolutionary biology, Earth system science, civil and environmental engineering, epidemiology, and political science. Two trainee representatives served on the executive committee to communicate trainee needs and feedback. The trainee representatives were nominated by their peers and served staggered 2-year terms to promote institutional memory. The four-member external advisory board included representatives from two outside academic institutions (Dr. Diane Pataki, University of Utah, and Dr. Darrel Jenerette, University of California, Riverside), a conservation non-profit (Dr. Nathan Gregory, Irvine Ranch Conservancy), and a joint powers research agency (Ken Schiff, Southern California Coastal Water Research Project). These external advisors were consulted to provide a real-world perspective in designing the program curriculum and training activities.

#### Program components

**Fig 2:**
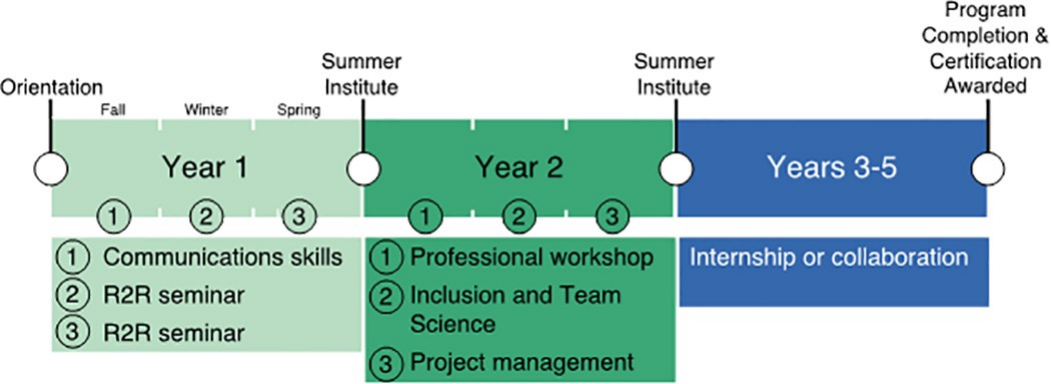
Final Ridge 2 Reef program timeline.

The R2R program consisted of four main components. The first included pre-curricular recruitment, admission, and orientation. The second component focused on structured curricular activities, including two years of formal coursework. Component three was a partnership/internship experience, and the fourth component comprised optional opportunities such as annual multi-day workshops called Summer Institutes, student-led and social events, funding opportunities, and career-related skills training. The program structure changed over time in response to feedback from trainees, faculty, and external advisors. The final program timeline is shown in Fig 2 and described in detail below.

The first-year curriculum aimed to improve and expand environmental research knowledge, interdisciplinary collaboration, and communication skills (Goal 1: Develop Scientific and Technical Knowledge; Goal 2: Promote Transferable Skills) with a Communication Skills course and series of seminars. In the Communication Skills course, trainees received instruction from a professional communication and presentation consultant to learn to communicate effectively with audiences outside of their disciplines and in non-technical language. Courses and seminars incorporated visiting speakers from sectors such as education, research, government, policy, non-profit, religion, public agencies, science communication, and industry who discussed career options and the skills and qualifications necessary to pursue those positions.

A three-course curriculum aimed to equip second-year trainees with leadership skills and opportunities for professional and career development, public engagement, professional certification, and career placement (Goal 2: Promote Transferrable Skills). The first course, Professional Workshop, included informational interviews with career professionals across sectors and professional development activities, such as certifications, tailored to individual students. The second course on Inclusion and Team Science explored the benefits and challenges of working in a collaborative environment and provided trainees with strategies to succeed in collaborative work. Finally, the Project Management course culminated in a collaborative project designed by the enrolled students.

At a time that was convenient to the trainees in program years 3–5, students both designed and participated in a partnership or internship, applying their training from the two years of courses to real-world issues (Goal 3: Build Partnerships). Partnership/internship requirements were flexible so the experience could be most effective in preparing an individual trainee for their specific field and career aspirations. The disciplinary focus of internships or partnerships varied based on trainees’ career and research goals.

Each program year, R2R hosted a themed Summer Institute in which trainees reinforced their technical skills, identified knowledge gaps in research fields, interacted across disciplines and cohorts, and developed their identities as researchers. Overall, R2R held four multi-day institutes, each with a specific theme: Climate and Life (2018), Microbes and Global Change (2019), Environmental Data Science (2021) and Program Success and Sustainability (2022). The 2020 Summer Institute was canceled due to the COVID-19 pandemic. The Microbes and Global Change and Environmental Data Science Summer Institutes were focused on building data analysis skills through writing code and completing analyses of ecological datasets in small groups.

#### Trainee support

To free up student time for training activities and encourage program participation (Goal 4: Broaden Participation), R2R offered financial support through competitive fellowships. After completing one year in R2R, trainees were eligible to apply for a one-year R2R fellowship that covered tuition and provided a stipend. Annual stipend levels were set according to NSF guidelines. The R2R fellowships were awarded by the program executive committee to trainees who demonstrated the potential to create new opportunities for research and training, support interdisciplinary collaborations and/or internship experiences, disseminate research outcomes broadly, and achieve academic or career goals.

Mentoring groups were established to provide professional support and promote program cohesion. The R2R program mentoring groups consisted of three or four trainees across different cohorts and one R2R faculty member. Senior trainees coordinated mentoring groups that met quarterly to discuss topics relevant to professional or academic development such as internship applications, grant writing techniques, and career progression.

## Methods

### Ethics statement

The formal evaluation of R2R was approved by the UCI Institutional Review Board (human subjects number 2017–3722). All participants provided informed verbal consent. The Institutional Review Board did not require or recommend obtaining written consent from participants. Prospective participants were provided with a study information sheet describing the evaluation process and goals. Consent to participate in the study was witnessed by project leaders and documented in a participant tracking sheet maintained by the project external evaluator. Consent was voluntary and could be revoked at any time with no penalty. No minors were involved in the study. The recruitment period for this study began on November 2, 2017, and ended on August 31, 2023.

### External program evaluation

The R2R executive committee contracted The Mark USA, Inc. (“The Mark”) to lead the formal program evaluation. Based in Irvine, California, The Mark is a professional evaluation firm with over 10 years of experience evaluating a broad range of academic and private sector programs funded by federal agencies such as the National Science Foundation and National Institutes of Health [17]. At the beginning and end of each academic year, The Mark administered quantitative evaluation surveys to assess progress toward achieving program goals. Assessment tools also included qualitative interviews of the trainees, faculty, and internship partners. Findings from the surveys and interviews were used to adjust program goals and guide the development of training activities. The results are discussed further and the raw data are publicly available [18].

#### Interview methodology

To understand the impact of different aspects of the program, program leaders conducted semi-structured interviews with trainees regarding their experience in the R2R program. Interviews took place at the end of each academic year starting with 2018–2019, when each trainee was interviewed separately. In the following years, trainees were interviewed in cohort-based focus groups, which varied in size from two to five trainees. Interviewers audio-recorded and transcribed the interviews. Transcripts were separated into idea units corresponding with the interview questions. The idea units were coded to identify themes related to program strengths, challenges, and recommendations for program improvements [18].

#### Survey methodology

Annual pre- and post-surveys were developed by The Mark in consultation with the R2R executive committee. Through that consultation, survey questions were specifically tailored to ensure that trainee responses were accurate metrics of progress toward R2R program goals. The pre/post survey method relies on self-assessment of knowledge and skills, which is a common practice for quantifying learning gains in educational research [19]. Although survey responses may be biased, this method provides quantitative data that complement qualitative data from interviews.

The surveys included Likert scale items and open-ended questions to assess goal areas and collect feedback on participants’ experiences in the program. Participants rated all Likert scale questions on five- or seven-point scales. The pre-survey asked questions that measured trainee knowledge and skills (including environmental policy and data analysis, communication, leadership, and mentoring skills), career interest, and collaborations in research and education-related activities. Pre-surveys were usually administered at the beginning of program year 1 for each cohort. However, all trainees in Cohort 1 took the pre-survey retrospectively at the end of program year 1, and two trainees in Cohort 2 and one trainee in Cohort 4 took the pre-survey retrospectively because they enrolled later in the evaluation study (Table 1). In the post-survey at the end of each academic year, participants were asked to rate their knowledge and skills again to assess annual changes in these areas, to report their collaborations over the year as well as their current career interest and knowledge, and to provide feedback on courses and program activities. Analyses performed on matched trainee responses include those respondents who completed their respective pre-survey and a later post-survey.

**Table 1:** Ridge 2 Reef cohort sizes and survey responses over five program years.

Year	2017–2018	2018–2019	2019–2020	2020–2021	2021–2022
	# Trainees	# Trainees	# Trainees	# Trainees	# Trainees
**Cohort 1**	9	9	6	5	1
**Cohort 2**		12	12	12	11
**Cohort 3**			8	7	5
**Cohort 4**				19	18
**Cohort 5**					7
**Total**	9	21	26	43	42
**Pre-survey responses**	7^a^	11^b^	4	14^c^	6
**Post-survey responses**	7	15^d^	12	29	29
**Post-survey response rate**	78%	71%	46%	67%	69%
**Matched responses**	7	14	10	26	26

^a^ Cohort 1 pre-surveys were retrospective at end of 2017–18

^b^ Includes two pre-surveys not taken until the following year

^c^ Includes one retrospective pre-survey taken mid-year

^d^ Includes two incomplete surveys from Cohort 2

### Communication skills course assessment

In addition to evaluating communication skills with pre- and post-surveys of trainees, the instructor for the Communication Skills course (Dr. Steven Allison) developed a rubric for pre- and post-assessment of presentation and writing skills. The rubric included 12 criteria for presentation skills, such as vocal performance, message clarity, use of supporting examples, and presentation structure [18]. Trainees submitted four-minute video lectures on a topic of their choosing at the beginning and end of the course. The instructor scored the video presentations according to the rubric, using a 0–3 scale in 2018 and a 1–7 scale in subsequent years where scores above 2 or above 5, respectively, indicated proficiency. The rubric for writing skills included eight criteria related to content, structure, and style. Trainees submitted 500-word public abstracts of their research that were scored pre- and post-course using the same scales as the presentation rubric.

### Data analysis

For pre- and post-survey data, we averaged Likert scores within eight skill or knowledge categories by trainee and survey year. The categories included disciplinary knowledge, interdisciplinary experience, global research perspective, communication skills, data analysis skills, leadership skills, mentoring skills, and career knowledge. Each category was represented by 4–15 unique questions [18]. To assess knowledge and skill development as trainees progressed through the R2R program, we tracked how average Likert scores for each category and cohort varied with year in program (“scores analysis”). Using matched trainee responses, we also computed gains in knowledge or skills by subtracting pre-survey Likert scores from post-survey Likert scores (“gains analysis”). We assessed the average gains across all cohorts at the end of each academic year (2018 to 2022). The gains analysis tested R2R program effectiveness over time.

For both scores and gains analyses, we fit survey data to generalized linear mixed models using the *glmmTMB* package in R, v1.1.9 [20]. For the scores model, cohort and year in program were fixed categorical effects with trainee ID as the random effect. For the gains model, cohort and calendar year were fixed categorical effects with matched trainee ID as the random effect. Model residuals were checked for normality and found to be normal or near normal based on the Shapiro-Wilk statistic. Because cohort effects and interactions with year were generally weak, we ran reduced models without the cohort factor for use in post-hoc hypothesis testing. We compared Likert scores from the pre-survey with scores from the post-surveys in subsequent years using *t*-tests corrected for multiple comparisons with the *emmeans* R package, v1.10.2 [21]. For the gains analysis, we assessed whether the model-estimated mean gains for each calendar year differed significantly from zero using *z*-tests.

To test learning gains in presentation and writing skills from the Communication Skills course, we analyzed rubric scores with a linear mixed-effects model using *glmmTMB*. Course year and pre- versus post-assessment were included as fixed effects, and trainee ID was included as a random effect, meaning that pre- and post-scores were paired for each trainee. Mean pre- and post-scores within each year were compared with post-hoc *t*-tests corrected for multiple comparisons. For all statistical tests, the threshold for significance was p < 0.05. R scripts for all analyses are available in [18].

## Results

### Goal 1: Develop scientific and technical knowledge

**Fig 3:**
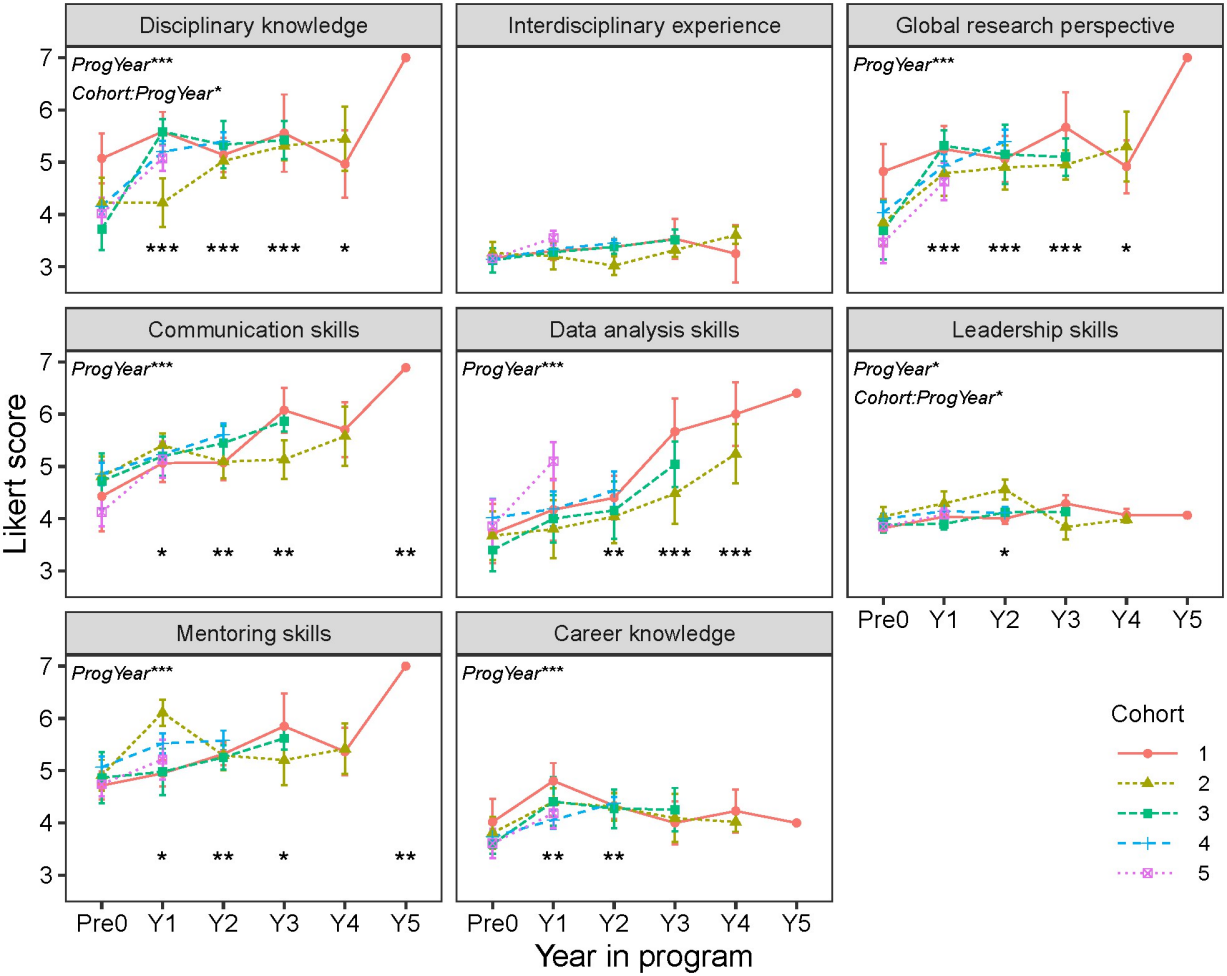
Self-reported Likert scores in eight skill and knowledge areas by cohort and year in the R2R program. Significant effects from linear mixed models are shown in the upper left corner of each panel. Asterisks at the bottom of each panel indicate significant differences for each year in program (Y1-Y5) between post-survey means and the pre-survey mean across cohorts (Pre0). Points indicate cohort means with standard error bars. Likert scales were 1–7 points except that interdisciplinary experience and leadership skills used a 5-point Likert scale and career knowledge included both 5- and 7-point scales. * p < 0.05; ** p < 0.01; *** p < 0.001.

**Fig 4:**
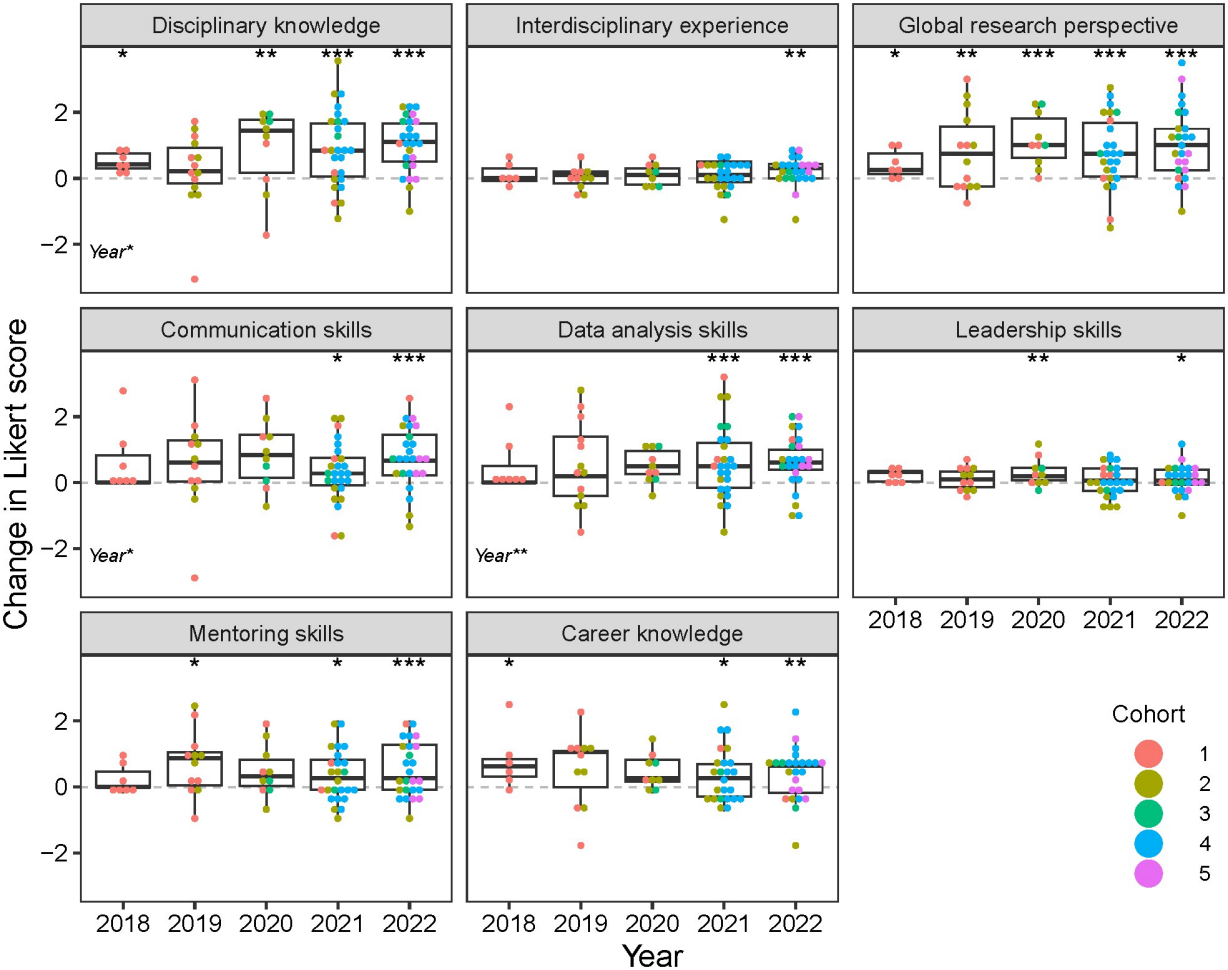
Change in Likert scores for eight skill and knowledge areas by calendar year. Change is computed as post-survey minus pre-survey for matched trainee responses. Significant effects from linear mixed models are shown in the lower left corner of each panel. Boxplots represent medians with interquartile ranges. Asterisks at the top of each panel indicate significant differences from zero for each year. * p < 0.05; ** p < 0.01; *** p < 0.001.

Evaluation data showed that trainees’ scientific and technical knowledge increased during the R2R program. Self-reported disciplinary knowledge grew with time spent in the program, although there was an interaction with cohort driven by a lack of change in Cohort 1 scores (Fig 3). However, matched surveys showed some disciplinary knowledge gains for Cohort 1 trainees in 2018 (Fig 4). Global research perspective showed a similar but stronger pattern of increase in both average Likert scores (Fig 3) and gains from matched surveys (Fig 4). Detailed statistical outputs are available in the Supplementary Material for the Likert scores analysis (S1 and S2 Tables) and gains analysis (S3 and S4 Tables).

Based on survey data, interdisciplinary experience did not change significantly as trainees progressed through the program (Fig 3). However, there was a significant gain observed across all cohorts in 2022 (Fig 4), suggesting that curriculum changes implemented later in the R2R program may have ultimately enhanced trainees’ interdisciplinary experience. Trainee interviews also supported this interpretation. In 2019, trainees from Cohorts 1 and 2 noted a lack of disciplinary diversity in R2R seminars, which limited opportunities for interdisciplinary collaborations and internships. However, by 2022, trainees noted that R2R allowed them to engage with a diverse community of scholars with shared goals, with one trainee commenting “I was really excited to join an interdisciplinary group of folks.”

### Goal 2: Promote transferable skills

Trainees made important strides in developing career-relevant transferrable skills. Likert scores for skills in communication, data analysis, and mentoring improved consistently with time in the R2R program (Fig 3). Leadership skills and career knowledge showed a different pattern, with Likert scores increasing at first and then leveling off after one or two years in the program. In addition, the trajectory of leadership skill development varied across cohorts (p = 0.045 for cohort: program year interaction). Note that only Cohort 1 and 2 trainees could participate in the program for more than three years, so we have limited statistical power to assess changes in skill levels of late-stage trainees. However, analysis of matched trainee surveys showed significant gains in all transferrable skills and career knowledge by 2022, the last year of the program (Fig 4). Significant gains were also reported for 2021, except for leadership skills, which showed a significant gain in 2020 but not 2021.

Trainee interviews corroborated the survey results on transferrable skills. From 2019 onward, trainees stated that skill-building activities in the R2R curriculum were beneficial for their professional development. In 2020, one trainee emphasized that the purpose of the program “is to provide additional training…to graduate students…who otherwise wouldn’t have the opportunities to gain those skills and experiences through their normal program.” Progress on these skills likely resulted from the Summer Institute on Microbiomes and Global Change offered in 2019 and the Summer Institute on Environmental Data Science offered in 2021. During interviews, trainees commented that the institutes were valuable for integration of scientific, technical, and professional skills to work on problems in small, interdisciplinary groups.

Rubric scores from the Communication Skills course independently confirmed the learning gains reported in trainee surveys. Presentation and writing skills improved significantly based on comparison of pre- versus post-assessment rubric scores (Table 2). Average post-scores greater than 2 in 2018 and greater than 5 in 2019–2021 indicated that most trainees achieved proficiency in communication skills. Furthermore, all 13 trainees who responded to the 2022 post-survey and enrolled in the Communication Skills course indicated that the course was very or extremely influential in building their communication skills.

**Table 2:** Mean (standard error) rubric scores for presentation and writing skills based on assignments from the R2R communication skills course.

Year	Presentation skills			Writing skills		
	Pre	Post	*t* (df = 52)	Pre	Post	*t* (df = 52)
2018^a^ (n = 9)	1.89 (0.13)	2.79 (0.05)	3.7***	1.79 (0.03)	2.67 (0.06)	4.5***
2019 (n = 5)	5.47 (0.48)	6.52 (0.18)	3.2**	3.73 (0.38)	6.03 (0.23)	8.8***
2020 (n = 11)	5.20 (0.18)	6.20 (0.20)	4.6***	3.42 (0.24)	6.40 (0.10)	16.9***
2021 (n = 6)	4.49 (0.43)	6.35 (0.30)	6.3***	4.08 (0.18)	6.40 (0.07)	9.7***

Asterisks indicate statistically significant improvements in post- versus pre-assessment skill levels.

** p = 0.002;

*** p < 0.001

^a^ The 2018 rubric used a 0–3 scale versus a 1–7 scale in 2019–2021.

### Goal 3: Build partnerships

Multiple lines of evidence show that R2R succeeded in building partnerships across disciplines and intuitions. The disciplinary focus of internships or partnerships varied from research and education to policy, communication, and fine arts across over 40 experiences with 37 different entities. These internships and collaborations included participation with eight non-profit organizations (e.g., Irvine Ranch Conservancy), 14 government agencies (e.g., National Aeronautics and Space Administration) from city to federal levels, 11 educational groups or institutes (e.g., Coastal Ocean Environment Summer School in Ghana), and four industry groups (e.g., IQ Air). Within these internships and collaborations, trainees worked on projects in a range of areas from communication, education, and research to conservation and policy. These experiences helped drive large increases in trainees’ knowledge and understanding of global research perspectives (Fig 4). Trainees who completed internships rated them as one of the most important experiences of their graduate careers. For example, comments during interviews included: “opened my eyes to possible options” and “one of the best things about the program.”

In the early program years, networking with professionals in science beyond UCI was limited to individuals (trainees and professionals) who could meet in person. Trainees expressed during interviews that they felt limited in their ability to find internships with partner organizations. Both graduate students and alumni lacked formal avenues to stay connected, for example through an alumni or research conferences. The external evaluator recommended that a directory of former alumni could benefit R2R students by creating new opportunities for communication and mentorship. Information such as post-graduate career pathways and job openings could be posted through the directory, allowing R2R participants to make connections with alumni in different fields within and outside of academia. In response to these needs, the R2R program launched a LinkedIn page in 2021.

During interviews, doctoral trainees highlighted a need for more flexible R2R requirements that could more easily align with specific graduate program requirements and individual dissertation research needs. As a result, starting with Cohort 2, internship requirements were relaxed and re-branded as a partnership requirement. This requirement was intentionally broadened so trainees and their faculty advisors could align internship experiences or collaborations with the specific needs of each trainee.

Benefits of external partnerships and collaborations were not only limited to students and the university. Interviews showed that external partners expanded their capacity for solving environmental problems by working with R2R trainees and faculty. Interviewees reported organizational benefits such as building a stronger connection with the university and an increased capacity to conduct research. Three partners noted that R2R internships brought individuals with a great deal of high-quality research experience into their organizations. For example, one partner mentioned that collaboration with R2R helped create a scientific foundation for managing water systems, which had an impact on achieving the organization’s mission. Another partner commented that their R2R intern helped obtain feedback from the community and determine if specialized projects should continue.

### Goal 4: Broaden participation

**Fig 5:**
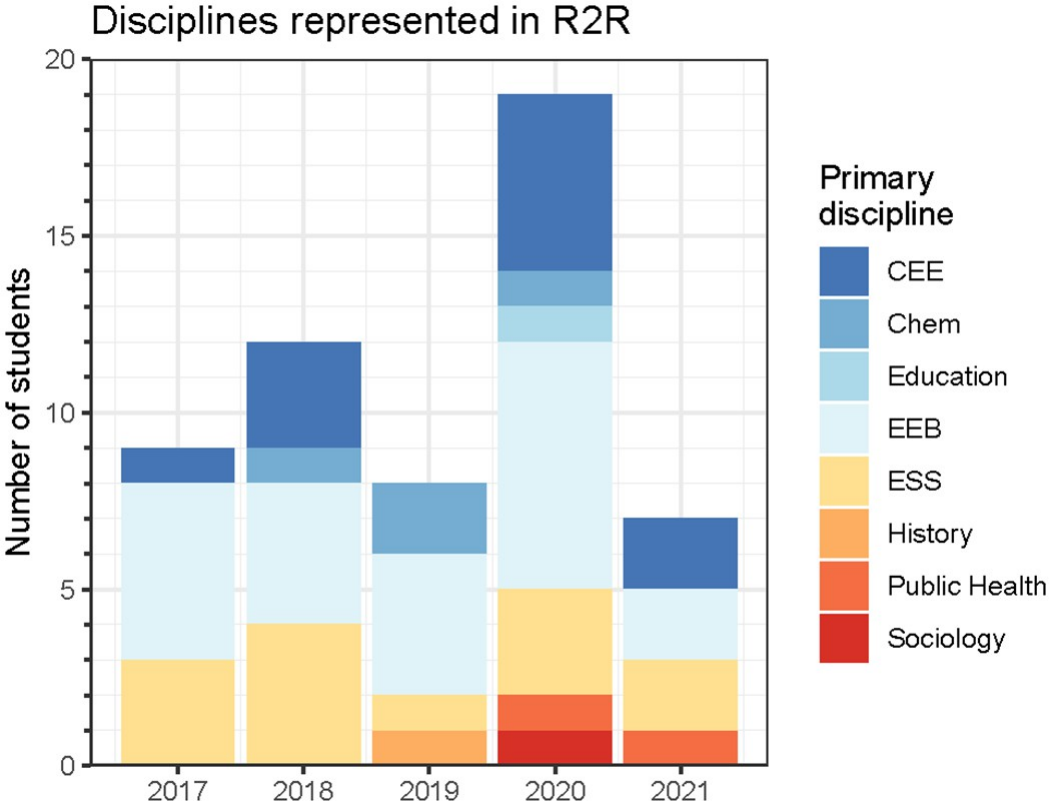
Disciplines represented in Ridge 2 Reef cohorts. CEE is civil and environmental engineering, EEB is ecology and evolutionary biology, and ESS is Earth system science.

Ridge to Reef achieved its goal to recruit and train a diversity of students. Cohorts ranged in size from 6 to 19 trainees, and disciplines varied between years (Fig 5). Most trainees came from STEM fields such as ecology and evolutionary biology, Earth system science, and civil and environmental engineering, while a smaller number of trainees came from outside STEM fields, including history and education. Program targets for broadening participation were set to reflect national demographics, with a goal of >50% identifying as females or non-binary and >32% identifying as underrepresented minorities (URM). By the end of the program, 69% of trainees responding to evaluation surveys were female and 46% were URM. As of spring 2022, 15 trainees had finished their degrees, and 3 trainees had withdrawn from UCI (2 trainees) or R2R (1 trainee) with the remaining 37 trainees on track to complete their degrees. Of graduated survey respondents (n = 11), all held professional or academic positions in STEM.

The R2R program began an internal mentoring initiative in program Year 3, which was intended to address interview feedback that highlighted a lack of faculty involvement, low diversity in faculty mentors, and limited interaction between R2R cohorts and across departments. During the initial roll-out of the mentorship program, mentoring groups included one faculty mentor, one peer mentor, and 2–3 additional trainees. Trainees and faculty were assigned to mentoring groups by the R2R academic coordinator. In its first year, the mentoring program received mixed reviews with suggestions that more structure and/or guidance for mentoring groups was needed. In its second year (program year 4) the mentoring program had a similar format, with added guidance on quarterly discussion topics provided by the academic coordinator to provide structure. The mentoring program continued to receive mixed reviews, with some mentoring groups being very satisfied with the program while others were dissatisfied. In the third iteration of the mentoring program, the academic coordinator sent out a survey to ask trainees about their goals for the mentoring groups and then assigned trainees to groups accordingly to improve mentorship compatibility and productivity within the groups.

Further, the perceived lack of disciplinary diversity within the R2R faculty (most were affiliated with ecology or Earth system science) posed a problem throughout the entirety of the program, leading to some trainees not feeling supported and a subsequent decline in both staff and student involvement within and across cohorts. Despite a dynamic combination of student trainees, the combined effect of low social interaction and lack of disciplinary representation initially limited trainees’ ability to participate in collaborations with other researchers.

Survey responses and interviews confirmed that fellowship support facilitated participation in R2R training activities as well as buy-in from faculty mentors. One trainee expressed that being on an R2R “fellowship [gave them] cushion to focus on research and expand [their] network,” and 18% of the 2022 post survey respondents indicated being awarded a grant or fellowship as their greatest achievement in the R2R program. Aside from funding for their students, faculty also realized benefits from R2R’s training model. The R2R curriculum was successful at developing students’ technical and professional skills, enabling faculty to focus on other trainee needs.

### Goal 5: Institutionalize success

In their interviews, trainees articulated how R2R created a successful model for addressing critical feedback on graduate training. Especially in the first two years of the program, trainees indicated that R2R courses were too time intensive. Trainees encountered difficulties balancing research, coursework, and teaching assistantships–a primary source of funding for some. In 2019 and 2020, the program was revamped to address these issues by dropping two disciplinary-focused courses and revising the second-year courses, resulting in the program structure that was described in the Introduction. Trainee responses to this curriculum redesign were positive overall, and trainees appreciated R2R leaders’ ability to make substantive changes to the program. The six required courses taken by R2R Cohorts 2–5 were maintained until the end of the program, indicating a shift towards a consistent and sustainable curriculum.

Another challenge addressed by R2R was communication about program requirements. After program year 1, trainees shared with the executive committee that they were unclear about expectations and opportunities for funding from the program. Early R2R cohorts observed that fellowship funding guidelines were ambiguous and confusing. As a result, application prompts were clarified and improved over time based on trainee feedback.

Trainees also expressed that program expectations appeared unstructured and unclear after joining the program. A lack of clarity and communication resulted in persistent confusion about program goals, expectations, and progression. These problems were ultimately alleviated by program leaders creating an R2R program handbook and hosting an orientation for new trainees. By year 5, once multiple cohorts had experienced the full curriculum, trainees viewed the program structure favorably, with one commenting that “I would say the structure of the program is really well done.”

## Discussion

The R2R program was designed to provide transferrable and interdisciplinary skill training, which is often not addressed in traditional graduate programs, to better prepare a diverse group of graduate students to face the inherently complex challenges that come with environmental problem solving. The program successfully trained students in communications, project management, and quantitative skills, among others, as evidenced by survey and interview responses. Although evaluation sample sizes were limited by the number of students who chose to enroll in the program and participate in surveys and interviews, we obtained enough responses to detect changes in all knowledge and skill categories based on pre- versus post-surveys.

### Goal 1: Develop scientific and technical knowledge

The R2R evaluation plan assessed disciplinary and interdisciplinary knowledge, demonstrating significant gains in these areas. Although we did not formally assess transdisciplinary knowledge, interdisciplinary training offered through the R2R program has helped students move towards transdisciplinary thinking, and R2R trainees have become ambassadors for transdisciplinarity. For example, a team of trainees designed and hosted the inaugural *Art + Ecology*: *Stories that Build Connections* Gala in spring 2022, bringing together artists and ecologists to re-envision ways of thinking about solutions to local environmental challenges. Related to that vision, the students spearheaded an effort to create an interdisciplinary art space at the Burns-Piñon Ridge Reserve, a University of California natural reserve in the Western Mojave Desert. This effort continued into the undergraduate sphere via a weekend of workshops in the new art space as well as a graduate course in art and ecology co-led by an R2R trainee. These outcomes demonstrate that R2R training is driving an institutional shift toward cultural attitudes and practices that support transdisciplinary scholarship. Such a shift is important because transdisciplinary approaches allow for co-creation of knowledge across sectors to generate outcomes that are greater than the sum of their disciplinary parts, such as the founding of new fields or concepts [22, 23].

### Goal 2: Promote transferable career-related skills

Overall, R2R trainees reported increased confidence in their skills and clarity in their career trajectories. The Communication Skills and Project Management courses, as well as internships, collaborative projects like this paper, and opportunities to put skills into practice during R2R events, helped trainees increase their knowledge and confidence in transferable skills not offered in their home programs. The communications component of the R2R program was very successful, with students reporting a significant boost in their ability to communicate effectively in oral and written contexts to lay audiences and professionals in and out of the student’s field of study. These learning gains were corroborated with assessment rubrics used by the Communication Skills course instructor.

The R2R program provided opportunities for trainees to receive structured, consistent mentoring and skills training outside of the traditional academic apprenticeship model under which the responsibility for training falls predominantly on the faculty advisor. These mechanisms are important because faculty vary substantially in their backgrounds, experience, and availability for mentoring [24, 25]. Training from R2R filled these gaps in faculty mentorship capacity, benefiting both the advisor and the student. During interviews, advisors commented that they appreciated how R2R provided funding, opportunities to network, and access to alternative career pathways for their students.

### Goal 3: Build partnerships

Throughout R2R, trainees developed partnerships with each other through coursework, workshops, and collaborative projects and with external partners through internships and other collaborations. These relationships increase UCI’s visibility in multiple communities and create opportunities for future career placement as well as collaboration. Partnerships also boost the relevance and impact of university research by ensuring that it addresses community needs. The R2R course on Inclusion and Team Science equipped trainees with the tools to center ethics and equity in their relationships with partners. Such training is crucial for avoiding extractive research practices and prioritizing communication and research co-design with partners from the very beginning of a project, a crucial element of transdisciplinary research [26]. These principles of research justice will carry forward in the new programs stemming from R2R (Table 3).

**Table 3:** UC Irvine programs that have been supported by advances from the R2R NRT training model.

Program	Funding	Description	Connection to R2R
Wildland-Urban Interface Climate Action Network	University of California Climate Action Initiative ($5.5M)	Develop a transdisciplinary model for climate action and land stewardship in southern California’s wildland-urban interface	R2R PI Allison is lead investigator; project builds on research themes from R2R
Center for Ecosystem Climate Solutions (2019–2023)	California Strategic Growth Council ($4.6M)	Supports state environmental management needs through data-driven science and technology with partners from government, nonprofit, and private sectors	Center Director Michael L. Goulden is R2R co-PI
Graduate Recruitment Cluster in Environmental Racism and Health Equity (2022)	UCI Graduate Division and matching funds from schools ($325K)	Recruitment and training of 20 graduate students in community-based research practices related to addressing environmental racism and health disparities	Adopts R2R communication skills course and recruitment plan; R2R PI Steven D. Allison is a co-PI on the cluster
CLIMATE Justice Initiative (2023)	NSF Cultural Transformations in the Geoscience Community ($7.5M)	Supports graduate student and post-baccalaureate training to center diversity, equity, and environmental justice in climate change research	Adopts R2R curriculum model; R2R PI Steven D. Allison is a co-PI on the project; PI Kathleen R. Johnson is an R2R faculty mentor
Masters in Conservation and Restoration Science (2017)	Fee-based professional master’s program	Trains masters’ students in the practice of conservation and restoration science across terrestrial and marine habitats	Shared program coordinator with R2R; shared courses for some trainees; research training seminar adopted from R2R
Newkirk Center for Science and Society (2001)	Endowment from the Newkirk family (~$250K/year)	Supports research, training, and events that explore the interface between the scientific community and societal needs	R2R PI Steven D. Allison is Director of the Newkirk Center since 2021; R2R recruitment practices and curriculum are helping to shape the Newkirk Graduate Fellows program

Partnerships built during the R2R program were maintained throughout the program, regardless of external factors including the COVID-19 pandemic. Although there was a substantial decline in social interactions among trainees from fall 2020 to spring 2021 in all cohorts, metrics of program success, such as number of partnerships and gains in knowledge and skills improved during 2021 and 2022. This progress suggests that R2R partnerships were strong enough that external collaborations and internships could resume as soon as pandemic restrictions were lifted.

### Goal 4: Broaden participation

Expanding diversity in both disciplines and participant demographics was key to achieving program goals. From program year 2 onwards, R2R met its diversity goals focused on inclusion of underrepresented groups. Although survey data only tracked participation by trainees self-identifying as URM, the recruitment and pedagogical practices adopted by R2R could also promote participation by students identifying as low-income, first generation, or people of color (POC). The R2R program did face some challenges with few trainees enrolling initially due to a lack of knowledge about the program on the UCI campus. The R2R website was still being developed and finalized which might explain some of these growing pains while interest from students, faculty, and departments was still being established.

Aside from achieving its goal of >32% URM participation, retention of all trainee groups—including URM students—exceeded 90% with only 3 of 55 trainees leaving the R2R program. As part of the recruitment strategy, R2R program leaders decided early on to emphasize building a supportive, inclusive community to which URM trainees would feel a sense of belonging. The R2R program prioritized high-quality training, community-building, and engagement with diverse partners to create an inclusive space for graduate education. Trainees appreciated these priorities during their time in the program, commenting positively on the importance of having a diverse community in creating a supportive environment and conducting interdisciplinary research. The emphasis on community building provided clear retention benefits for URM students and the institution. Fellowship support and the prestige associated with participation in NSF’s flagship traineeship program may have also contributed to high retention rates. Going forward, these outcomes will likely attract URM recruits into new programs developed from R2R.

### Goal 5: Institutionalize success

Graduate students contribute to the research and educational mission of universities, so benefits they experience can have positive impacts on institutions [27]. The R2R program aimed to institutionalize successful elements and disseminate its training model to other institutions (Goal 5: Institutionalize Success). Program leaders recognized graduate students as essential for making connections across research teams, providing a foundation for transdisciplinary research and education. A highly skilled and confident community of graduate trainees tackling environmental grand challenges is a tremendous asset for any university. By fostering this community, R2R added value to UCI’s human capital as well as the workforce into which trainees enter. Moreover, programs like R2R that set and achieve goals in minority representation can help create a more diverse environmental workforce.

The term “culture of improvement” was coined during focus groups with R2R trainees to describe how trainee feedback informed program decision-making. We define culture of improvement as the willingness and ability of a higher education program to directly integrate assessment evidence into decisions on program and curricular structure and teaching practices [28, 29]. For R2R, the culture of improvement was particularly strong in the intentional use of feedback and (re)design of program elements. This culture of improvement likely contributed to significant gains in skill development observed across cohorts in 2021 and 2022 (Fig 4).

There is emerging evidence that R2R has begun to shift institutional attitudes toward interdisciplinary scholarship. Some of this shift is occurring through new and ongoing programs that have adopted R2R curriculum and ideas (Table 3). One of the NRT program’s main goals was to develop bold new models of graduate education with benefits that extend beyond individual grants in time and scope. At UCI, R2R has expanded the capacity for research and education through multiple avenues. Trainees have generated over 40 publications in 34 different journals and given dozens of presentations on their research. Papers that trainees authored were published in disciplines ranging from social ecology to hydrology and marine biology in journals including *Nature Sustainability*, *Proceedings of the National Academy of Sciences*, and *Journal of Problem Based Learning in Higher Education*. The full list of publications can be accessed on the NSF Public Access Repository (https://par.nsf.gov/search/term:1735040). Beyond these traditional metrics of research output, teams of faculty have been successful in leveraging R2R ideas and training elements to build new programs, including several that are externally funded (Table 3). In all cases, these programs have included R2R program faculty and adopted elements of the R2R training model such as courses, research ideas, and recruitment plans.

### Lessons learned from R2R

1. Focus on student outcomes—It was imperative to keep the program focused on student outcomes, particularly regarding required courses. Initial classes were not program-specific but were drawn from existing course offerings of program faculty. Those courses left some program and student needs unmet. Consequently, the R2R curriculum was revised to be more flexible and student-driven, allowing trainees to apply their knowledge and skills in new contexts, further developing their career adaptability and confidence.
2. Conduct rigorous program evaluation—It was very beneficial to have multiple mechanisms for program evaluation. A mix of pre/post surveys, interviews, and feedback from the trainee representatives on the executive committee ensured that program leaders quickly learned which elements of the program were not working well. Regular assessment is key to identifying specific challenges and making programmatic changes, ultimately fostering the “culture of improvement” and adoption of more effective courses or activities.
3. Prepare students for collaboration—Students should be trained in collaboration and team research before expecting them to engage in partnerships or internships. Early cohorts that jumped into such partnerships without sufficient training had a harder time succeeding in projects with partners. Also, additional training responsibilities were pushed onto the partner, reducing the time available for research and collaboration with the trainee.
4. Diversify learning outcomes—Events such as the Summer Institutes should be structured around broad learning outcomes that go beyond traditional research and technical skills. Workshops proved more successful and well-received by trainees when time for socializing among peers, recreation, and networking was allotted in the schedule. Building inter-personal connections and self-confidence is crucial for trainee success in the program. Evaluations also revealed that throughout the program, trainees developed partnerships with each other through collaborative projects, coursework, and workshops including the Summer Institutes.
5. Build community—Graduate school can feel isolating, and especially during the Covid pandemic, social events were an invaluable aspect of the program for trainees. The R2R program not only organized program social events but also supported trainees in designing their own events. Social events varied in size and form, ranging from pastry and coffee hours to symposia, lunches, seminars, and the *Art + Ecology* Gala. Providing a space and structure for socializing allowed trainees to build community, resulting in new collaborations and partnerships while fostering teamwork skills. Trainees also gained perspectives on different fields, collaborations, and ways of thinking that they may not have otherwise encountered.

### Implications for graduate education

Any graduate program, regardless of focus, can benefit from the student-focused culture of improvement that emerged from the R2R training model [10]. When implemented as the foundation of a program, this educational perspective can strengthen training for students and positively impact their careers. The commitment to academic, professional, and cultural diversity throughout all aspects of R2R ensured an atmosphere of consideration for multiple perspectives and ideas. The integration of surveys and rapid, feedback-driven changes to program structure ensured that students’ needs were respected and aligned with the program goals.

A dynamic program like R2R is rare within academia, which is often bogged down by constraints of traditional graduate programs including institutional legacies, outdated measures of student outcomes, lack of willingness to change, and lack of interdisciplinary training [10, 30, 31]. Intentional collection of qualitative and quantitative data on stakeholder engagement within the program, with the intention to listen and respond, allowed R2R to foster a culture of improvement. Other programs seeking to replicate the R2R training model should consider investing in regular program evaluation and involving trainees in program decision-making. Taking trainee feedback seriously enhanced skill development and satisfaction with the program (Figs 3 and 4).

Graduate programs that establish a culture of improvement will better prepare the next generation of researchers to address complex societal problems. Many trainees enter graduate school with an aim to address the difficult problems that society faces; for our trainees, that often means climate change and other environmental problems [32, 33]. Societal problems like climate change are, by definition, transdisciplinary in scope [2]. For academic research to remain relevant in a complex world, graduate training must reflect the transdisciplinary and constantly changing nature of the world’s problems [10].

Other programs can implement practices to promote a culture of improvement, thereby addressing some of the shortcomings in traditional graduate education. The R2R program demonstrated that soliciting feedback from current students, faculty, and staff is essential for making effective changes to curriculum and training activities. Whether it be the redesign of offered courses, building new partnerships on and off campus, or creating opportunities for students and faculty of different disciplines to interact, graduate programs can benefit from staying student-focused and constantly striving to improve.

## Supporting information

S1 Table ANOVA statistics (Type 2 Wald χ^2^) for Likert scores analysis. (DOCX)

S2 Table Post-hoc *t* statistics for mean Likert score difference from pre-survey. (DOCX)

S3 Table ANOVA statistics (Type 2 Wald χ^2^) for Likert score gains analysis. (DOCX)

S4 Table *Z*-scores for average gain in Likert score by calendar year. (DOCX)
