# Supplementary material for: From Ridge 2 Reef: An interdisciplinary model for training the next generation of environmental problem solvers
Source: PLoS One. 2024 Dec 19;19(12):e0314755. doi: 10.1371/journal.pone.0314755 (PMC11658476; doi:10.1371/journal.pone.0314755)
Supplement: S1 Table — (DOCX) [file pone.0314755.s001.docx]

Table S1. ANOVA statistics (Type 2 Wald χ^2^) for Likert scores analysis

| Category | Cohort |  |  | Year |  |  | C:Y |  |  |
| --- | --- | --- | --- | --- | --- | --- | --- | --- | --- |
|  | χ^2^ | df | P | χ^2^ | df | P | χ^2^ | df | P |
| Disciplinary | 3.4 | 4 | 0.489 | 52.0 | 5 | <0.001 | 21.5 | 10 | 0.018 |
| Interdisciplinary | 1.4 | 4 | 0.844 | 9.4 | 4 | 0.052 | 15.1 | 10 | 0.129 |
| Global | 4.2 | 4 | 0.380 | 52.7 | 4 | <0.001 | 7.8 | 10 | 0.649 |
| Communication | 3.0 | 4 | 0.565 | 30.9 | 5 | <0.001 | 9.5 | 10 | 0.484 |
| Data skills | 2.1 | 4 | 0.722 | 41.5 | 5 | <0.001 | 10.4 | 10 | 0.403 |
| Leadership | 1.0 | 4 | 0.915 | 11.5 | 5 | 0.042 | 18.6 | 10 | 0.045 |
| Mentoring | 2.4 | 4 | 0.655 | 27.3 | 5 | <0.001 | 13.3 | 10 | 0.210 |
| Career | 1.2 | 4 | 0.875 | 20.4 | 5 | 0.001 | 7.9 | 10 | 0.639 |
