# Supplementary material for: From Ridge 2 Reef: An interdisciplinary model for training the next generation of environmental problem solvers
Source: PLoS One. 2024 Dec 19;19(12):e0314755. doi: 10.1371/journal.pone.0314755 (PMC11658476; doi:10.1371/journal.pone.0314755)
Supplement: S2 Table — (DOCX) [file pone.0314755.s002.docx]

Table S2. Post-hoc *t* statistics for mean Likert score difference from pre-survey

| Category | Y1 |  | Y2 |  | Y3 |  | Y4 |  | Y5 |  |
| --- | --- | --- | --- | --- | --- | --- | --- | --- | --- | --- |
|  | *t* | P | *t* | P | *t* | P | *t* | P | *t* | P |
| Disciplinary  df = 131 | 5.03 | <0.001 | 5.50 | <0.001 | 4.41 | <0.001 | 2.95 | 0.017 | 2.02 | 0.173 |
| Interdisciplinary  df = 125 | 2.11 | 0.122 | 2.30 | 0.078 | 2.01 | 0.150 | 1.00 | 0.688 |  |  |
| Global  df = 130 | 5.44 | <0.001 | 6.35 | <0.001 | 4.05 | <0.001 | 2.85 | 0.023 | 1.39 | 0.493 |
| Communication  df = 128 | 3.00 | 0.015 | 3.71 | 0.002 | 3.67 | 0.002 | 2.41 | 0.073 | 3.19 | 0.008 |
| Data skills  df = 129 | 2.46 | 0.064 | 3.41 | 0.004 | 4.84 | <0.001 | 3.89 | 0.001 | 2.38 | 0.078 |
| Leadership  df = 128 | 2.27 | 0.101 | 2.89 | 0.021 | 0.74 | 0.871 | 0.09 | 1.000 | 0.39 | 0.974 |
| Mentoring  df = 127 | 2.96 | 0.017 | 3.38 | 0.005 | 3.11 | 0.011 | 1.20 | 0.611 | 3.14 | 0.010 |
| Career  df = 125 | 3.39 | 0.005 | 3.81 | 0.001 | 2.54 | 0.053 | 0.76 | 0.862 | 0.133 | 1.000 |
