# Supplementary material for: From Ridge 2 Reef: An interdisciplinary model for training the next generation of environmental problem solvers
Source: PLoS One. 2024 Dec 19;19(12):e0314755. doi: 10.1371/journal.pone.0314755 (PMC11658476; doi:10.1371/journal.pone.0314755)
Supplement: S3 Table — (DOCX) [file pone.0314755.s003.docx]

Table S3. ANOVA statistics (Type 2 Wald χ^2^) for Likert score gains analysis

| Score category | Cohort |  |  | Year |  |  | C:Y |  |  |
| --- | --- | --- | --- | --- | --- | --- | --- | --- | --- |
|  | χ^2^ | df | P | χ^2^ | df | P | χ^2^ | df | P |
| Disciplinary | 8.5 | 5 | 0.133 | 11.5 | 4 | 0.022 | 3.9 | 6 | 0.693 |
| Interdisciplinary | 9.7 | 5 | 0.084 | 6.8 | 4 | 0.147 | 7.9 | 5 | 0.161 |
| Global | 6.5 | 5 | 0.264 | 3.4 | 4 | 0.497 | 1.3 | 6 | 0.973 |
| Communication | 4.4 | 5 | 0.495 | 12.8 | 4 | 0.012 | 5.4 | 6 | 0.498 |
| Data skills | 11.0 | 5 | 0.050 | 17.1 | 4 | 0.002 | 1.5 | 6 | 0.958 |
| Leadership | 4.6 | 6 | 0.463 | 5.7 | 4 | 0.225 | 5.3 | 6 | 0.512 |
| Mentoring | 2.2 | 5 | 0.823 | 6.5 | 4 | 0.164 | 9.1 | 6 | 0.166 |
| Career | 6.9 | 5 | 0.225 | 2.7 | 4 | 0.618 | 8.4 | 6 | 0.213 |
