# Supplementary material for: From Ridge 2 Reef: An interdisciplinary model for training the next generation of environmental problem solvers
Source: PLoS One. 2024 Dec 19;19(12):e0314755. doi: 10.1371/journal.pone.0314755 (PMC11658476; doi:10.1371/journal.pone.0314755)
Supplement: S4 Table — (DOCX) [file pone.0314755.s004.docx]

Table S4. *Z*-scores for average gain in Likert score by calendar year

| Category | 2018 |  | 2019 |  | 2020 |  | 2021 |  | 2022 |  |
| --- | --- | --- | --- | --- | --- | --- | --- | --- | --- | --- |
|  | *z* | P | *z* | P | *z* | P | *z* | P | z | P |
| Disciplinary  n = 83 | 2.34 | 0.019 | 0.77 | 0.443 | 3.00 | 0.003 | 5.00 | <0.001 | 5.77 | <0.001 |
| Interdisciplinary  n = 74 | -0.21 | 0.833 | -0.16 | 0.875 | 0.98 | 0.328 | 1.81 | 0.070 | 3.19 | 0.001 |
| Global  n = 82 | 1.98 | 0.048 | 3.28 | 0.001 | 3.64 | <0.001 | 3.97 | <0.001 | 5.63 | <0.001 |
| Communication  n = 80 | 0.54 | 0.586 | 0.49 | 0.622 | 1.58 | 0.114 | 2.32 | 0.020 | 4.52 | <0.001 |
| Data skills  n = 81 | -0.24 | 0.812 | 0.54 | 0.587 | 1.79 | 0.073 | 4.19 | <0.001 | 4.27 | <0.001 |
| Leadership  n = 80 | 1.52 | 0.130 | 1.51 | 0.130 | 3.08 | 0.002 | 1.27 | 0.204 | 2.00 | 0.046 |
| Mentoring  n = 79 | 0.34 | 0.733 | 2.49 | 0.013 | 1.35 | 0.176 | 2.27 | 0.023 | 3.62 | <0.001 |
| Career  n = 75 | 2.08 | 0.038 | 1.45 | 0.146 | 1.87 | 0.061 | 2.22 | 0.026 | 3.11 | 0.002 |
